# Membranous Croup and Its Treatment

**Published:** 1875-10

**Authors:** Bedford Brown


On Membranous Croup and its Treatment.—*By Bedford Brown, M.D.*—Setting aside all theories in regard to the specific character of croup as unsustained either by arguments or by facts, those pathological processes combining to complete the disease known as membranous croup comprise, primarily, engorgement; secondarily, inflammation; and, lastly, exudation on the mucous membrane of the larynx and trachea; or, rather, the destruction and sloughing or exfoliation of the involved epithelium, and then the membranous exudation. This process of epithelial exfoliation is truly one of the most important of all those elements entering into the formation of the disease. Without this preliminary arrangement it would not be possible for the last and most important stage—that of exudation—to occur.

Hence, when the destruction and exfoliation of the epithelial coat have taken place, the basement membrane underneath is laid bare; it can no longer secrete mucus, but becomes the theatre of those important actions consisting in the exudation of plastic material from the exposed vessels, which rapidly assumes the form and consistency of membranes.

Not in all, but in a very large proportion of the cases of tonsillitis coming under our observation, in greater or less degree these identical processes occur and may be observed at any time.

In such cases the affected tonsil first becomes engorged, then highly inflamed, then one or more white or gray patches, sometimes larger than a shilling, appear on its surface. These may disappear and reappear several times before resolution. These cases are usually denominated ulcerative, and, at other times, diphtheritic. In reality they are neither, but of a true exudative character with an innocent type of disease. If such exudations were situated in the larynx or trachea, they would then become matters of infinite moment. In tonsillitis of this character, until the inflammatory action subsides, we may observe this membranous exudation, though removed by local applications, return every day. Its removal is usually followed by bleeding.

The same destruction by exfoliation of the epithelial coat occurs here as in membranous croup. Thus, while the epithelial coat exists in its perfection with unimpaired functions, there can be no membranous formation. This fact is one of paramount importance in the pathological history of croup and its therapeutic management.

Bearing on this point there is another pathological question of infinite importance. It is whether membranous croup is a simple inflammatory affection, or a specific disease. Universal experience in the profession unites in establishing the opinion that by appropriate treatment the exudation may be prevented. Hence the conclusion that this form of croup is a simple form of inflammation. Under intense inflammation, the epithelium ceases to perform its function of secreting mucus. There is an utter suspension of action, and consequently a complete absence of all moisture on the epithelial surface. There is no relief of the engorgement and blood-stasis, and this delicate coat sloughs, leaving the basement membrane denuded, with its injected vessels laden with plastic blood, when exudation results. In simple tonsillitis with limited exudation we have ocular demonstration of the fact that this exudation, when left undisturbed, continues to grow in proportion to the area of the destruction of the epithelium, and also of the fact that during a high state of inflammatory action the epithelial coat so destroyed cannot be repaired until resolution begins. Hence the successive crops of exudation in membranous croup, diphtheria, and tonsillitis, while inflammation continues.

It would appear that in all local affections of an exudative character, morbid action must reach a certain point, must pass only through certain stages, and must be surrounded with favorable circumstances, to complete the process of exudation. When this process is interrupted, either the normal secretion of the part affected takes place, or purulent formation is substituted. Thus, in the case of incised wounds, the adhesive or plastic form of inflammation is often through slight influences converted into the purulent or serious products either by local or by general influences. In membranous croup we desire to convert the plastic form of the inflammatory products into the mucous before exudation has taken place, and, if possible, into the purulent after that has occurred. Thus, if the epithelial cells coating the inflamed surface should be made to pour out their peculiar secretion there can be no plastic exudation, and these delicate bodies are saved from destruction.

*Treatment of the Inflammatory Stage.*—Whatever agents will cause a free secretion of mucous in this stage of croup, will prove the best means of preventing the last or exudative stage.

Iodide of potassium is unquestionably one of the most prompt and certain stimulants of the mucous secretion in our possession. With this valuable property it combines an alterative power over inflammations of mucous membranes, which gives it peculiar adaptation to the treatment of the inflammatory stages of this affection. The object in using it is to cause the mucous membrane of the bronchial system to pour out its secretions copiously, with a view of saving the epithelial coat from being destroyed, and preventing exudation. So long as the fever and inflammation are active, the cough clear and metallic in character, the voice hoarse, the iodide may be used energetically and freely. This remedy, to be of service in this disease, must be used in heroic doses, repeated at intervals of one or two hours. Time is a precious consideration in the treatment of croup, and to insure success the system must be saturated with the drug as speedily as possible. The remarkable sedative influence exerted by this preparation over inflammations of the respiratory tract gives it additional value in the treatment of croup. Its sedative power in the turbulent and labored respiration of asthma and emphysema, in dry catarrh, and in kindred affections, is unequalled by that of any other drug for permanent effect. In addition to this, it is especially adapted to the respiratory diseases of childhood. The action of iodide of potassium over the respiratory tract begins with the Schneiderian membrane, and embraces the mucous surface of the mouth, the entire glandular system pertaining to salivation, the pharynx, larynx, trachea, and bronchial membrane. The normal secretions peculiar to all these surfaces are greatly augmented by its agency. Indeed, its remarkable powers as an expectorant are far from being understood or appreciated.

In the inflammatory stages of croup it may be advantageously combined with the bicarbonate and bromide of potassium, and glycerin, which latter has valuable expectorant properties. . . .

Under the free and energetic use of the iodide in these affections, either alone or in the above combination, when the system is fully saturated with the drug the quantity of salivary and mucous secretion poured forth is sometimes astounding. This is true of croupal, tonsillitic, and catarrhal affections. In cases of tonsillitis with intense injection and tension of mucous surface, and attended with great dryness and want of moisture, the iodide will usually stimulate free secretion from the fauces, to the infinite relief of the local affection.

In a considerable proportion of the cases treated by the iodide of potassium in large doses, there was free and copious salivation, but without any of the peculiar inflammation of the salivary glands resulting from the use of mercury. . . . . .

*Treatment of the Exudative Stage.*—After exudation has been fully established a different system of treatment becomes necessary.

The tincture of the chloride of iron, combined with the chloride of ammonium and chlorate of potassium, are the only general reliable means in this stage. They act best when dissolved in glycerin and water. Glycerin is always a valuable agent in croup, as it is one of the few articles which invite moisture to the inflamed surface in the form of sero-mucous secretion.

Thus, when the symptoms of orthopnœa become more permanent, and the fever declines without corresponding improvement, this treatment should be instituted vigorously and without delay. The system must be saturated with the remedies as rapidly as possible ; consequently they should be given every hour. . . . .

The chloride of iron is not given here, as in diphtheria and its kindred diseases, as a corrective of blood-poisoning, but for its remarkable influence over local disease of a diffuse inflammatory character attended with either exudation, effusion, or extravasation. When absorbed into the circulation in sufficient quantity, it exerts a marked influence on the capillary vessels in the remotest part of the system, contracting their calibre, reducing dilation, correcting engorgement, and arresting exudation. —*Philadelphia Medical Times.*

